# Catheter-Directed Thrombolysis in Acute Iliofemoral Deep Vein Thrombosis with or without Stenting: A Case Series

**Published:** 2018-10

**Authors:** Ali Mohammad Haji Zeinali, Yaser Jenab, Hamid Ariannejad, Seyed Ebrahim Kassaian, Mohammad Alidoosti, Hassan Aghajani, Mohammad Reza Zafarghandi

**Affiliations:** 1 Tehran Heart Center, Tehran University of Medical Sciences, Tehran, Iran.; 2 Sina Hospital, Tehran University of Medical Sciences, Tehran, Iran.

**Keywords:** *Venous thrombosis*, *Postthrombotic syndrome*, *Stents*

## Abstract

Iliofemoral deep vein thrombosis (IFDVT) is a potentially devastating condition comprising a quarter of all cases of lower extremity DVT. It can lead to serious consequences such as pulmonary embolism, limb malperfusion, and post-thrombotic syndrome (PTS), which is a chronic sequela of IFDVT. We herewith present 18 IFDVT cases managed with catheter-directed thrombolysis at our hospital. Nine of these patients underwent stenting of the involved iliac veins. The remaining 9, who did not receive stenting, had a residual stenosis of more than 50% in the common femoral or iliac veins following the procedure. Based on a final residual stenosis of less than 50% in the iliac veins, we had 9 successful (patients with stenting) and 9 unsuccessful procedures (patients without stenting). In subsequent follow-ups at a median follow-up of 39.5 months, using the Villalta score, while only 2 out of the 9 patients who underwent stenting suffered PTS, 4 patients among the other 9 patients comprising the non-stenting group developed PTS. Our results support the notion that stenting might have a role in decreasing the PTS risk in patients undergoing catheter-directed thrombolysis.

## Introduction

Deep vein thrombosis (DVT) is a potentially life-threatening condition. Iliofemoral deep vein thrombosis (IFDVT) is defined as a thrombus involving any part of the iliac and/or common femoral veins, with or without extension to the inferior vena cava (IVC). Thrombosis involving the iliofemoral veins accounts for about a quarter of all cases of lower extremity DVT and has a worse prognosis than proximal DVT not involving the common femoral or iliac veins. It is associated with a high risk of pulmonary embolism, limb malperfusion, and post-thrombotic syndrome (PTS) when compared with DVT cases occurring below the knee. Patients with IFDVT are less well studied than those suffering from other forms of DVT and may benefit more from acute intervention.^1^^-^^3^ Herein, we present 18 such cases of IFDVT, managed with catheter-directed thrombolysis (CDT) at our hospital.

## Report of the Cases

In a 6-year period, from 2011 to 2017, among patients with a clinical picture suggestive of DVT who were admitted to Tehran Heart Center, 18 (8 male and 10 female) patients with an established diagnosis of IFDVT (Table 1) were treated invasively in a 1-step procedure (using catheter thrombectomy/thrombolysis/balloon-venoplasty, with or without stent placement) in our catheterization laboratory. In all the patients, DVT symptoms had been present for a period of fewer than 2 weeks by the time of presentation. The diagnosis was made clinically at first and was later confirmed based on ultrasonography. The median (25^th^, 75^th^ percentile) age of the patients was 43 (33, 60) years. Fourteen thromboses were left-sided, and the remaining 4 were right-sided. Three patients had dyspnea, which pulmonary computed tomography angiography confirmed as acute pulmonary embolism. Five patients had a history of recent hospitalization, including 1 with a history of a major orthopedic surgery and 2 with childbirth. Four of the female patients had been receiving hormone therapy (Table 1). The patients had a median (25^th^, 75^th^ percentile) creatinine value of 0.78 (0.63, 0.9) mg/dl. Ipsilateral popliteal vein cannulation under ultrasonography guidance was used to access the DVT lesion in all 18 patients. The access was from the left popliteal vein in 14 patients and from the right popliteal vein in the remaining 4. Retrievable IVC filters were implanted in 12 patients; 8 of these filters were successfully retrieved after the procedure. The median (25^th^, 75^th^ percentile) thrombolysis duration was 67.5 (40, 97) hours. The median (25^th^, 75^th^ percentile) administered heparin dosage from the side of the popliteal sheath was 500 (475, 500) U/h. As for the thrombolytic agent during CDT, 15 patients received streptokinase, 2 received alteplase, and the remaining 1 received reteplase. Eleven out of the 18 patients, who had CDT, underwent balloon angioplasty using peripheral balloon catheters for thrombus fragmentation and 1 patient underwent rotational thrombectomy with Aspirex thrombectomy (Straub Medical, Switzerland) (Table 2). 

The degree of lysis was measured using venography following thrombolysis with CDT but before stent deployment. After thrombolysis, no patient had complete lysis of the thrombus with CDT, 7 had successful partial (50%–99%) lysis, and 11 (<50%) patients had unsuccessful lysis. Nine out of the 18 patients underwent stenting of the involved iliac veins: sinus-venous stents were used in 7, the Maris Plus stent in 1, and the Epic Absolute stent in 1. All the patients without stenting (9 patients) had residual stenoses of more than 50% in the common femoral or iliac veins. Based on the final residual stenosis of the iliac veins of less than 50%, there were 9 successful (patients with stenting) and 9 unsuccessful procedures (patients without stenting). No patient expired during hospitalization. Major bleeding was reported in 9 patients: 3 had puncture-site hematoma and the remaining 6 experienced a significant drop in the hemoglobin value by more than 2 g/L, which prompted blood transfusion. There was no intracranial bleeding. Two patients developed minor bleeding: 1 epistaxis and the other hemoptysis (Table 2).

**Table 1:** Patient characteristics*

Age (y)	43 (33–60)
Sex	
Male	8 (44.4)
Female	10 (55.6)
DVT Side	
Left	14 (77.8)
Right	4 (22.2)
Hospitalization / immobilization	5 (27.8)
Major surgery	1 (5.6)
Childbirth	2 (11.1)
Creatinine level (mg/dl)*	0.78 (0.63–0.9)

DVT, Deep vein thrombosis

* Data are presented as n (%) or median (25th-75th percentile)

**Table 2:** Clinical features, management, and outcomes*

Venous Approach	
Right popliteal vein	4 (22.2)
Left popliteal vein	14 (77.8)
IVC filter placement	12 (66.7)
IVC filter retrieval after the procedure	8 (44.4)
Thrombolytic duration (hr)	67.5 (40–97)
Heparin dosage (U/h)*	500 (475–500)
Thrombolytic Drug Type	
Streptokinase	15 (83.3)
Alteplase	2 (11.1)
Reteplase	1 (5.6)
Type of Additional Method	
Balloon venoplasty	11 (61.1)
Thrombectomy	1 (5.6)
Stent placement	9 (50.0)
Procedure Result	
Successful	9 (50.0)
Unsuccessful	9 (50.0)
Bleeding (during 10 days post procedure)	
Major	9 (50.0)
Minor	2 (11.1)

IVC, Inferior vena cava

* Data are presented as n (%) or median (25th-75th percentile).

**Table 3:** Follow-up results

	DVT Side	Thrombolytic Infusion Duration (hr)	Stent Use	CDT Result	FU Time (mo)	VCSS	Villalta Score	PTS
Case 1	Left	34	Yes	Successful	12	0	0	No
Case 2	Left	80	Yes	Successful	21	9	14	Yes
Case 3	Left	40	Yes	Successful	23	4	4	No
Case 4	Right	100	No	Unsuccessful	23	2	2	No
Case 5	Left	40	Yes	Successful	24	0	0	No
Case 6	Left	96	Yes	Successful	25	0	0	No
Case 7	Left	90	No	Unsuccessful	25	0	1	No
Case 8	Left	40	Yes	Successful	32	2	2	No
Case 9	Left	141	No	Unsuccessful	39	17	24	Yes
Case 10	Left	90	No	Unsuccessful	40	0	0	No
Case 11	Left	45	No	Unsuccessful	40	Died	-	-
Case 12	Left	112	No	Unsuccessful	65	4	4	No
Case 13	Left	23	No	Unsuccessful	66	9	17	Yes
Case 14	Right	70	Yes	Successful	68	4	11	Yes
Case 15	Right	46	Yes	Successful	73	Died	-	-
Case 16	Left	65	No	Unsuccessful	76	4	5	Yes
Case 17	Left	123	Yes	Successful	78	0	0	No
Case 18	Right	62	No	Unsuccessful	80	18	27	Yes

DVT, Deep vein thrombosis; CDT, Catheter-directed thrombolysis; FU, Follow-up; VCSS, Venous clinical severity score; PTS, Post-thrombotic syndrome

Prior to CDT, all the patients received thorough explanations about the risks and benefits of the procedure and written informed consent was obtained from all of them. 

The patients were followed-up once at a median duration of 39.5 months. The follow-ups included recording the symptoms and signs of PTS as defined by the Villalta scale and the venous clinical severity score (VCSS). During the follow-up period, 2 patients died: 1 had successful CDT and the other one had an unsuccessful result. Out of the remaining 16 patients, 6 were identified as positive for PTS (Villalta score >4) and the other 10 patients were either symptom-free or scored below the diagnostic range for PTS based upon the Villalta scale. Comparisons of the follow-up findings with the procedure results demonstrated that only 2 patients out of the 9 patients with successful CDT for IFDVT developed PTS, while 4 patients among the other 9 patients comprising the unsuccessful group suffered PTS (Table 3).

## Discussion

In the present study, our clinical assessment of the patients (n=18), including the consideration of risk factors according to the Wells criteria in addition to the presenting symptoms and signs, led to the diagnosis of IFDVT. The associated risk factors of DVT in our cases were old age, history of trauma, postoperative state, prolonged immobility, obesity, hormone therapy, childbirth, and smoking.

Given the high variability in the clinical manifestations of acute DVT, it is recommended that the Wells score be drawn upon to establish the pretest probability of the presence of DVT. The Wells score, however, cannot assist in the differentiation of patients who may benefit from endovascular intervention or thrombolysis.^4^

We used Doppler ultrasonography to confirm the diagnosis for the patients who had a high probability of IFDVT. Even though venography is the gold standard for the diagnosis of DVT, ultrasound is usually a more practical option. The sensitivity and specificity of ultrasound for detecting proximal DVT is about 95%.^3^

The May–Thurner syndrome (MTS) is an anatomical condition resulting in the compression of the common venous outflow tract of the left lower extremity.^5^ This is probably because the left common iliac vein passes between the right common iliac artery and the lumbar vertebrae, where it is subject to arterial pulsation and constant pressure. In the majority of our cases (14 out of 18), DVT occurred in the left side. This finding is consistent with the literature, which notes a higher prevalence of left-sided DVT in patients with isolated DVT.^6^ In all of our 14 patients who presented with left-sided DVT, following CDT, the common iliac vein origin had significant residual stenosis, which was compatible with the MTS. The characteristic MTS lesion was diagnosed with venography following thrombus clearance. These lesions are defined by the stenosis of the proximal left common iliac vein, including the portion underlying the right common iliac artery, as well as the presence of significant venous collateral vessels. 

In 12 of our patients who had either extension of the thrombus to the IVC or symptoms of concomitant pulmonary embolism, to reduce the risk of the migration of clots to the heart and lungs during CDT, we deployed the OPTEASE retrievable IVC filter. Following the procedure, 8 filters were successfully removed, 2 filters were not retrieved because of the presence of trapped clots within the filter, 1 was not retrieved based on the clinical decision of the treating physician, and in 1 case attempts to retrieve the IVC filter were unsuccessful due to filter tilting. Of the latter 4 patients, during the follow-up, 2 developed PTS with Villalta scores of 24 and 17, 1 was dead, and 1 was identified as not having PTS with a Villalta score of 0.

CDT with additional balloon dilatation can yield better results in some cases of IFDVT as suggested by Zhang et al.^7^ Previous research has suggested that when there is little success through the course of CDT, ballooning might increase the amount and rate of success.^8^ Adjunctive mechanical thrombectomy, balloon angioplasty, and stenting can be done at the discretion of the interventionist.^9^ In our case series, 11 patients underwent balloon angioplasty during the CDT procedure and 1 patient received rotational thrombectomy with Aspirex thrombectomy at the discretion of the interventionist, who sought to accelerate the process of thrombus removal and stent placement. 

The current study is an overall report on the treatment of 18 individual cases managed in our center for IFDVT. Considering that this is a case series, no randomization was done and no control groups were present. In our case series, each patient was considered individually at the time of treatment. The main considerations for stent deployment or otherwise for each patient at the time of treatment were age and affordability of the stents.

Bleeding is the main complication of CDT. Significant bleedings are usually limited to the site of the venous puncture.^9^ In the CaVenT trial, 5 (3%) patients with CDT experienced major bleeding. The high rate of major bleeding (50%) in our study could be attributed to a long thrombolytic infusion of 67.5 hours (40–97 h) compared with that in the CaVenT study (2.4 d). Another reason could be the infusion of streptokinase in most of our patients, as opposed to alteplase in the CaVenT study.^10^

The most frequent complication of DVT is PTS, which is a serious and debilitating condition resulting from chronic venous insufficiency following lower extremity DVT.^11^ Patients who have chronic iliac vein obstruction are especially susceptible to severe PTS.^12^ PTS is thought to develop after DVT due to venous hypertension.^12^ Symptoms of PTS usually occur within 3 to 6 months after DVT, but they can manifest themselves even up to 2 years following DVT.^11^ The Villalta PTS scale has been devised to standardize the diagnosis and grading of the severity of PTS. It consists of 5 symptoms and 6 signs-including pain, edema, induration, changes in skin color, and venous ectasia. Each item is scored from 0 to 3, with a higher score indicating a greater degree of severity. A total score above 5 predicts the presence of PTS.^9^ PTS is a particularly common outcome following IFDVT probably due to inability to form collateral veins around the obstructed segment.^9^ One study found that IFDVT was the strongest predictor of PTS, more so than recurrent ipsilateral DVT.^9^^, ^^13^ Despite optimal anticoagulation, more than 30% of patients with a history of symptomatic DVT will develop symptomatic PTS-likely due to chronic venous occlusion, suboptimal collateralization pathways, and venous valvular dysfunction.^4^ It is well established that PTS has a significant negative impact on a patient’s quality of life.^4^ Options for the treatment of IFDVT include systemic anticoagulation, systemic thrombolysis, and pharmacomechanical CDT. In recent years, the efficacy of CDT for reducing the incidence of PTS in patients with DVT has been investigated by large clinical trials.

In the CaVenT trial, CDT was shown to significantly reduce the risk of PTS when used in conjunction with anticoagulation therapy for patients with proximal DVT.^10^ In this trial at 24 months, 41.1% of the patients treated by CDT presented with PTS. In our case-series, 6 out of the 18 patients devolved PTS during the follow-up.

Another study suggested that in patients with IFDVT, the use of CDT as an adjunct to anticoagulation therapy, compared with anticoagulation alone, resulted in better quality of life and fewer physical limitations.^3^^, ^^10^^, ^^14^ The ATTRACT trial evaluated the use of pharmacomechanical CDT for DVT and, in contrast to the aforementioned studies, reported that the additional pharmacomechanical CDT was associated neither with a reduction in the overall occurrence of PTS nor with a significant difference in quality of life among the patients despite a reduction in the severity of PTS. The investigators concluded that the addition of pharmacomechanical CDT for the treatment of proximal DVT had little clinical benefit.^15^

Regarding our patients, only 2 out of the 9 patients with successful CDT (with stent placement) developed PTS, whereas 4 among the other 9 patients comprising the unsuccessful group (without stent placement) suffered PTS. In the CDT group of the CaVenT trial, the frequency of PTS was not different between the unsuccessful, partial, and complete lysis and the distribution of percent lysis was similar in the patients with and without PTS. In this trial, only 15% of the patients received venous stents. However, stenting in patients with residual stenosis after CDT might further decrease the risk of PTS.

## Conclusion

Patients with acute iliofemoral deep vein thrombosis are still at risk of post-thrombotic syndrome during follow-up. Stenting might have a role in decreasing the post-thrombotic syndrome risk in patients undergoing catheter-directed thrombolysis.

## Notes:

***This paper should be cited as: ***Haji Zeinali AM, Jenab Y, Ariannejad H, Kassaian SE, Alidoosti M, Aghajani H, Zafarghandi MR. Catheter-Directed Thrombolysis in Acute Iliofemoral Deep Vein Thrombosis with or without Stenting: A Case Series. J Teh Univ Heart Ctr 2018;13(4):186-190.
